# Improvement in peritoneal dialysis exit-site infection rates in an integrated health care system in the United States

**DOI:** 10.3389/fneph.2026.1825353

**Published:** 2026-09-17

**Authors:** Max T. Hukill, Andrea M. Yeung, Talha H. Imam

**Affiliations:** 1 Kaiser Permanente Bernard J. Tyson School of Medicine, Pasadena, CA, United States; 2 Department of Nephrology, Southern California Permanente Medical Group, Fontana Medical Center, Fontana, CA, United States

**Keywords:** end-stage kidney disease, exit-site infection, integrated health care system, peritoneal dialysis, peritonitis

## Abstract

**Introduction:**

Exit-site infections (ESIs) are a significant complication of peritoneal dialysis (PD) due to their potential to progress to peritonitis, leading to higher rates of morbidity, mortality, and PD dropout. ESI rates and trends have not been generally recorded or reported by established registries. To address this gap, we studied data from ten Kaiser Permanente Southern California (KPSC) hospitals over the last decade to report yearly PD ESI rates and examine trends over time.

**Methods:**

This is a historical cohort study using data provided by the Renal Registry of Southern California Permanente Medical Group (SCPMG) for ten KPSC hospitals. Each hospital provided quarterly data from 2014–2023 with number of patients and ESI rate, reported as episodes per 100 patient-months. We modeled quarterly ESI rates across all centers (2014–2023) using a Tweedie generalized linear mixed model with calendar year and program size as fixed effects, accounting for repeated measures within center.

**Results:**

The overall mean ESI rate across the study period was 0.86 episodes per 100 patient-months (95% CI: 0.68–1.05). The observed mean ESI rate was 1.26 (95% CI: 0.98–1.60) episodes per 100 patient-months in 2014, compared with 0.72 (95% CI: 0.42–1.07) in 2023. Each additional calendar year was associated with a 6.4% relative decrease in ESI rate (rate ratio [RR] 0.94, 95% CI 0.89–0.99, *p* = 0.011), corresponding to an estimated 45.1% decline in ESI rate over the study period (95% CI: 12.8–65.4%). Program size was not significantly associated with ESI rate after adjustment for calendar year (RR 0.76 per 100-patient increase, 95% CI 0.52–1.11, *p* = 0.15).

**Conclusion:**

We found a significant decline in ESI rates across ten KPSC hospitals from 2014 to 2023. This decline was not explained by growth in program size, suggesting that other factors—such as evolving infection prevention practices, staff training, or care coordination—may have contributed, though our data do not permit us to test these mechanisms directly. We encourage prospective or mechanism-specific studies to explore which factors are driving these improvements.

## 1 Introduction

Peritoneal dialysis (PD) plays a major role in the management of patients with end-stage kidney disease (ESKD). PD is a significantly less expensive modality than hemodialysis (HD) (1), and studies have shown that patients on PD have a higher perceived quality of life than that of patients on HD (2).

However, technique failure and PD dropout remain major challenges to the utility of PD in the treatment of ESKD. PD-related peritonitis is the most frequent reason for PD catheter removal and patient dropout from PD (3, 4). Exit-site infections (ESIs), with or without tunnel infections, have the potential to lead to PD peritonitis. The relationship between ESIs and peritonitis was first noted in the 1980s, when it was found that patients with an ESI were more likely to develop peritonitis than patients without an ESI (5). A strong association between ESIs and subsequent PD peritonitis persisted up to 60 days after initial diagnosis of the incident ESI (6).

Peritonitis rates have been well documented in the literature. Prior multicenter published reports of peritonitis rates include the Peritonitis Organism Exit-Site Tunnel Infections (POET) clinical monitoring system developed by Baxter Healthcare. This report captured 4, 028 peritonitis episodes among 9, 655 patients across 35 United States (US) and 26 Canadian centers between 1988 and 2004; they reported overall peritonitis rates of 0.37 episodes per patient-year among US patients. Results from the 2020 Peritoneal Dialysis Outcomes and Practice Patterns Study (PDOPPS), involving 7, 051 adult PD patients in 209 facilities across seven countries, showed that PD peritonitis rates varied amongst countries. The lowest rates occurred in US patients with a rate of 0.26 episodes per patient-year (7). Those rates proved to be higher than the most recent PDOPPS 2020 published rates, reflecting improvements in peritonitis prevention strategies over time (7). The International Society of Peritoneal Dialysis (ISPD) published an updated guideline in 2022 that recommended that overall peritonitis rates should not exceed 0.4 episodes per year at risk (8).

Unlike peritonitis rates, patterns and trends of ESI rates remain significantly understudied. ESI rates have not been generally recorded or reported by large registries like the Australia and New Zealand Dialysis and Transplant Registry (ANZDATA) and the US Renal Data System (USRDS) (9). Some limited studies have reported rates ranging from 0.06 to 0.42 episodes per year (9–16), but these were controlled, small-scale trials not representative of real-world situations. One larger study from Lin et al. (2020) involved 1, 133 patients over one year and found an ESI rate of 0.13 episodes per year (12). A Portuguese study reported an ESI rate of 0.41 episodes per year with an average of 726 patients over two years (15).

Since no ESI rates have been reported from large multicenter studies or registries, we decided to review data from Renal Registry of Southern California Permanente Medical Group (SCPMG) over the last 10 years. We sought to calculate and report a 10-year cumulative PD ESI rate for the Kaiser Permanente Southern California (KPSC) hospitals and analyze associated patterns over time.

## 2 Materials and methods

### 2.1 Data source and study population

This is a historical cohort study using quarterly data provided by the Renal Registry of SCPMG for ten KPSC hospitals. Nine hospitals reported data throughout the full study period between 2014–2023; one hospital began reporting data in 2018 and was included in the study from that year onward. Of 382 eligible hospital-quarters, 7 (1.8%) had no recorded program size or ESI rate and were excluded as missing data; the pattern and handling of missing data are detailed in the **Supplementary Material (Supplementary Figure 1A)**.

### 2.2 Outcome definitions

*Exit-site infection rates* were calculated and reported at the hospital-quarter level as one episode per patient-months at risk. This format is consistent with geometric control (g-control) chart methodology commonly used in quality improvement for monitoring rare adverse events, in which the interval between successive occurrences is tracked (17). To facilitate comparison with published literature on ESI rates, we converted this value to episodes per 100 patient-months. For pooled rates, the 95% confidence intervals are generated by a location-cluster bootstrap (centers resampled with replacement, 5, 000 replicates).

*Program size* was recorded as the number of patients treated per quarter and was analyzed separately as a model predictor (see Statistical Modeling, below). Program size was not used in the calculation of ESI rate itself, as independent patient-months and program size data were not both available (see Limitations).

### 2.3 Exit-site infection definitions

All catheters were placed with advanced laparoscopy techniques in all KPSC hospitals with pre-insertion antibiotics (18). Per the International Society of Peritoneal Dialysis (ISPD), ESI was defined as the presence of purulent discharge with or without erythema of the skin at the catheter epidermal interface. Only these cases were cultured and included in our study. Cases with isolated tunnel infections have been extremely rare. Those with only erythema, tenderness, or swelling were not counted as an ESI and were excluded from the study. Negative culture results from ESIs with purulent discharge have been very uncommon in our program, but these cases were counted as ESIs. The definition and reporting practice remained stable across all hospitals and throughout the 10-year period.

The ISPD 2023 Catheter-Related Infection Recommendations revised and clarified the definitions for ESI diagnosis and classification, but did not establish formal definitions for “repeat, “ “relapsing, “ or “recurrent” ESI, unlike the definitions available for PD peritonitis (9). The ISPD does, however, use the term “refractory” ESI, which is defined as failure to respond to 2 weeks of effective antibiotic therapy and appropriately intensified exit-site care, or 3 weeks for ESI due to *Pseudomonas* (9). Hence, we recorded all ESI episodes as separate individual events.

Following diagnosis, care of the exit site and ESI treatment was carried out according to ISPD guidelines in all KPSC PD centers (9). Daily treatment of the exit site involved washing, topical antibiotic—either mupirocin or gentamycin, sometimes with additional sodium hypochlorite 0.057% spray (Anasept™)—and a sterile dressing. Most centers did not use prophylactic mupirocin. However, we also note that variations in care likely occurred across KPSC PD centers, consistent with the results of the PDOPPS (7).

### 2.4 Statistical modeling

We modeled ESI rate using a Tweedie generalized linear mixed model, appropriate for the semicontinuous, right-skewed distribution of ESI rates with an exact point mass at zero (17.9% of hospital-quarters). The model included fixed effects for calendar year and program size, allowing us to evaluate the association between program size and ESI rate while directly accounting for the concurrent temporal trend, rather than assessing either variable in isolation. A random intercept and slope for calendar year were included by hospital, allowing each hospital rate of change over time to vary; this specification was selected over a simpler random-intercept-only model based on residual diagnostics and a likelihood ratio test (**Supplementary Figure 1B**).

*Model diagnostics and sensitivity analyses.* All data cleaning, visualization, and analysis were performed in R (R Core Team) using the tidyverse collection of packages (19). We assessed model fit using simulation-based residual diagnostics (DHARMa) (20) and evaluated multicollinearity between fixed effects using variance inflation factors. We conducted two sensitivity analyses: (1) a model with a nonlinear (quadratic) term for program size, (2) a model excluding the hospital with delayed reporting. Full results are reported in the **Supplementary Material (Supplementary Tables 1, 2**) (21).

## 3 Results

### 3.1 Descriptive ESI rates and program sizes

ESI rates for all ten hospitals over the study period are shown in **Figure 1**. The overall mean ESI rate across the study period was 0.86 episodes per 100 patient-months (95% CI: 0.68–1.05). Mean rates declined from 1.26 (95% CI: 0.98–1.60) in 2014 to 0.72 (95% CI: 0.42–1.07) in 2023 (**Table 1**, **Figure 1**). As an illustrative approximation of cohort size at the beginning and end of our study period, nine KP hospitals collectively contributed 631 patients per quarter in 2014 (sum of quarterly averages), while ten KP hospitals collectively contributed 933 patients per quarter in 2023 (**Table 1**). We visualize the patient contribution of each KP hospital over time in **Figure 2**. Some practices grew larger over the course of the study (such as Hospital H) whereas others maintained a similar-sized practice throughout (such as Hospital C).

**Figure 1:**
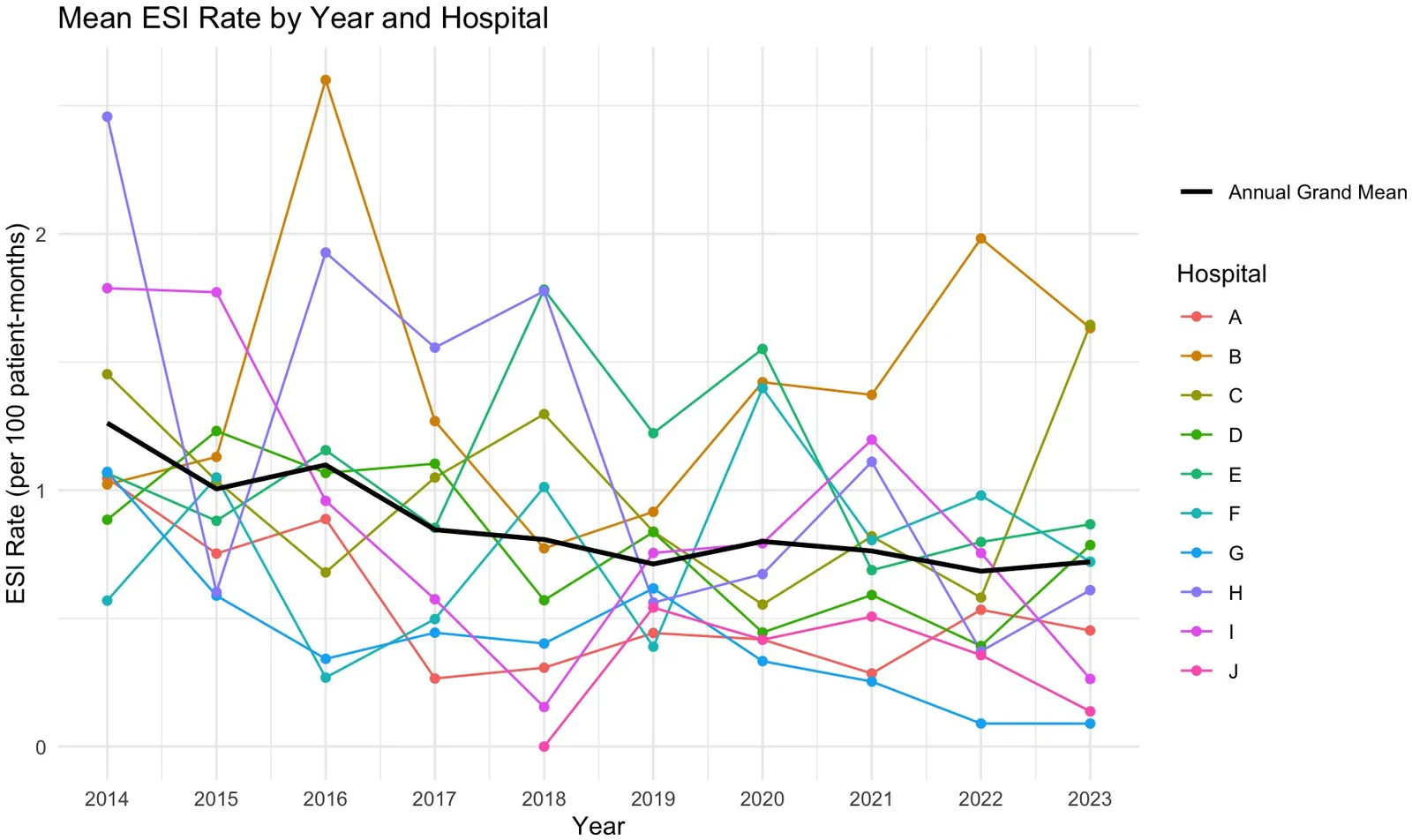
Exit-site infection rates vs. time for all ten KP hospitals, overlayed with average ESI rate (black). Time is reported as yearly data (an average of quarterly data) for the decade under study, and ESI rates are represented as episodes per 100 patient-months.

**Table 1:** Year-level ESI rate summary, 2014-2023.

Year	Quarters observed	Centers reporting	Quarters without infection (% of total observed)	Mean program size (patients)	ESI rate per 100 patient-months (95% CI)
2014	32	9	12.5	71.3	1.26 (0.98-1.60)
2015	35	9	14.3	74.5	1.00 (0.79-1.25)
2016	36	9	22.2	79.4	1.10 (0.69-1.58)
2017	36	9	22.2	87.4	0.85 (0.59-1.10)
2018	37	10	21.6	95.3	0.86 (0.49-1.24)
2019	40	10	20	95.3	0.71 (0.57-0.87)
2020	40	10	20	97.9	0.80 (0.54-1.08)
2021	39	10	5.1	94.2	0.77 (0.54-0.99)
2022	40	10	17.5	93.9	0.68 (0.42-1.03)
2023	40	10	22.5	93.3	0.72 (0.42-1.07)
2014-2023 (Overall)	375	10	17.9	88.8	0.86 (0.68-1.05)

Rate is expressed as episodes per 100 patient-months (mean of hospital-quarter rates, 95% CI from hospital-cluster bootstrap, 5, 000 replicates).

**Figure 2:**
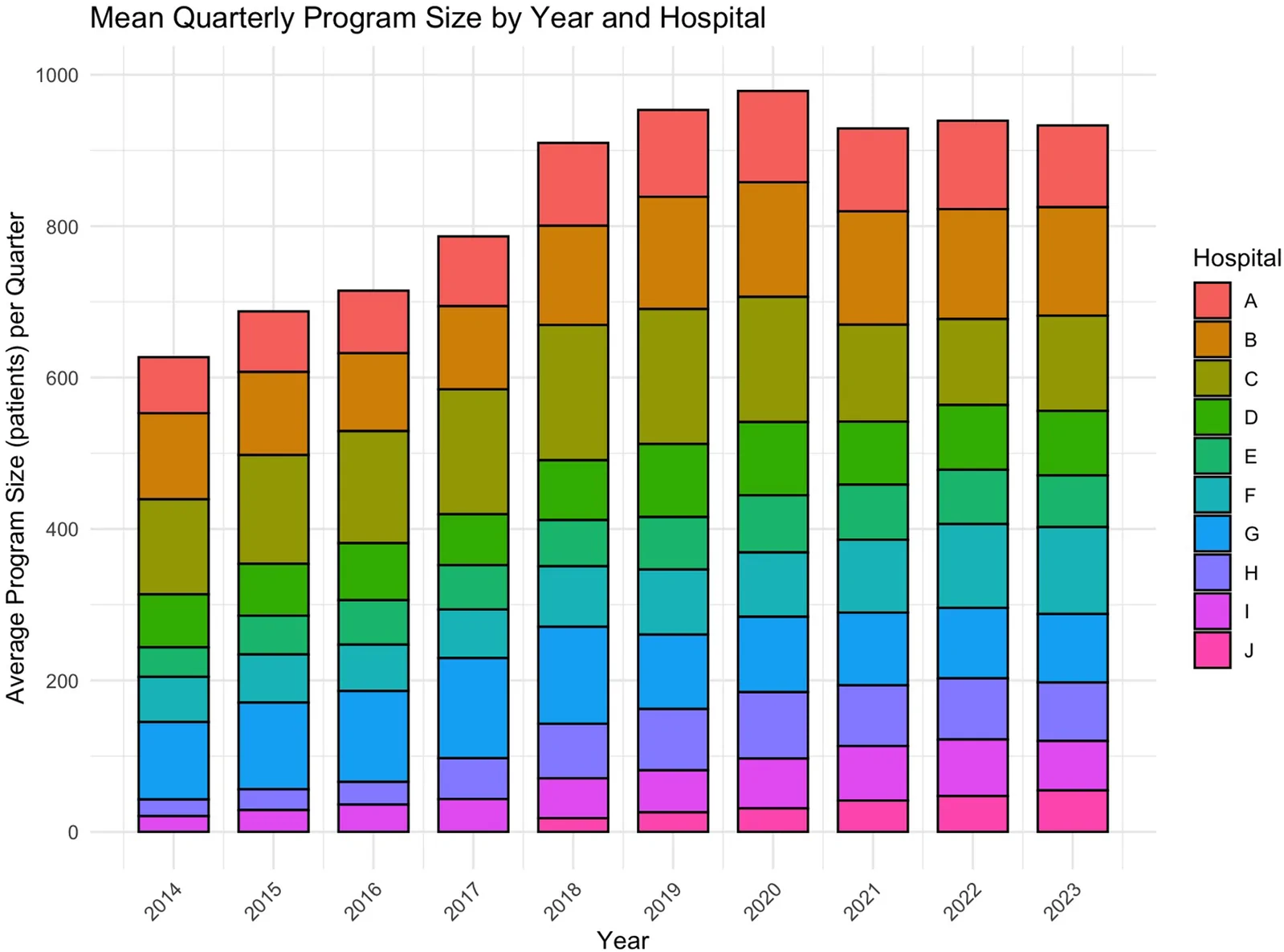
Number of PD patients in each KP hospital over time. Each colored section of a given bar represents the average number of PD patients for a particular KP hospital over the four quarters of that year (as opposed to an annual sum).

### 3.2 Primary model

In the Tweedie generalized linear mixed model, each additional calendar year was associated with a 6.4% relative decrease in mean ESI rate (rate ratio [RR] 0.94, 95% CI 0.89–0.99, *p* = 0.011), corresponding to an estimated 45.1% decline in ESI rate over the ten-year study period (95% CI: 12.8–65.4%) (**Table 2**). Program size was not significantly associated with ESI rate after adjustment for calendar year (RR 0.76 per 100-patient increase, 95% CI 0.52–1.11, *p* = 0.15) (**Table 2**). Variance inflation factors for calendar year and program size were low (VIF = 1.39 for both terms), indicating that this null finding was not attributable to collinearity between the two predictors. Further information about the model selection and diagnostics as well as sensitivity analyses can be found in the **Supplementary Material**.

**Table 2:** Change in exit-site infection rates per 1-year increase and per 100-patient increase, based on primary model results.

Term	RR (95% CI)	*p*-value
Calendar year (per 1-year increase)	0.936 (0.889-0.985)	0.011
Program size (per 100-patient increase)	0.755 (0.517-1.105)	0.148

Estimates from a Tweedie generalized linear mixed model (log link), fit by maximum likelihood (glmmTMB), with fixed effects for calendar year and program size and a random intercept and slope for calendar year by dialysis center. RR = rate ratio.

### 3.3 Study population and missing data

Missing data did not cluster significantly by center (*χ*²=6.5, *p* = 0.76) but did cluster significantly by year (*χ*²=22.0, *p* = 0.012), driven predominantly by the first study year (4 of 7 missing hospital-quarters occurred in 2014), consistent with expected reporting ramp-up at study onset rather than informative missingness. Nine hospital reported throughout the full study period; one hospital began reporting in 2018 and contributed 21 hospital-quarters. The final analytic sample comprised 375 hospital-quarters (**Supplementary Figure 1A**).

## 4 Discussion

This study adds to the existing literature by reporting trends in ESI rates across multiple hospitals over a substantial period of time. This study is the largest retrospective analysis of data that has been published thus far on ESI rates. Our data analysis demonstrates a statistically significant decrease in mean ESI rates at ten KPSC hospitals over the last decade (2014–2023), with quantitative estimates for decreased infections as a function of time. Specifically, each additional calendar year was associated with a 6.4% relative decrease in ESI rate (rate ratio [RR] 0.94, 95% CI 0.89–0.99, *p* = 0.011), corresponding to an estimated 45.1% decline in ESI rate over the study period (95% CI: 12.8–65.4%). Program size was not significantly associated with ESI rate after adjustment for calendar year (RR 0.76 per 100-patient increase, 95% CI 0.52–1.11, *p* = 0.15).

The impetus of this statistically significant decrease in ESI rates has multiple plausible explanations. The ISPD guidelines have played a major role in educating physicians and advancing care for patients with ESKD (9). A multidisciplinary approach is used, starting with patient education about catheter care prior to surgery. Surgeons then use advanced laparoscopic techniques for PD catheter placement, along with pre-insertion antibiotics (18). The exit site is created with minimal trauma and an optimized exit-site position so that it is not located under abdominal folds, along belt lines, or facing downward. Very close monitoring of exit sites is performed by PD nurses post-surgery. Subsequently, enhanced exit-site care protocols, including sterile techniques, hand hygiene, strong emphasis on proper immobilization of catheters, and the use of topical antibiotic cream, could be facilitating improved ESI rates. At many centers, patients also use daily topical sodium hypochlorite (Anasept ^®^) along with antibiotic creams (22). If any trauma, irritation, or redness is noted, 3% saline is frequently used as well as cautery when granulation tissue is present at exit-site edges. At all KPSC hospitals, nurses provide continuous support and regular follow-up to monitor patient practices and adherence, with retraining as needed. More frequent follow-up on patients, a collaborative environment, and a surveillance system with tracking of infections help identify potential problems with the exit site that may progress to infection, thereby assisting in early intervention.

Furthermore, experience with treating PD has cumulatively grown over time from two standpoints. First, providers have had more years of clinical practice treating PD patients over the past decade. Second, KPSC hospitals have witnessed an increase in the PD patient population. These factors may have contributed to greater cumulative clinical experience amongst nephrologists and nurses, which in turn may help explain the observed decline in ESI rates. While this study did not show that increasing PD cohort size was associated in lower ESI rates, this effect has been demonstrated by Htay et al., who showed that centers with higher proportions of dialysis patients treated with PD had significantly higher odds of peritonitis cure (23).

From a modeling perspective, our diagnostics indicated significant heterogeneity in the rate of ESI decline over time between distinct KPSC hospitals (random slope variance, SD = 0.06; likelihood ratio test versus a random-intercept-only model, *p* = 0.004). While most centers showed declining trends broadly consistent with the population-level estimate, one center (B) showed a trend in the opposite direction (**Figure 3**). Given the limited number of hospitals in this analysis (*n* = 10), center-specific trend estimates carry substantially more uncertainty than the population-level estimate and should be interpreted as hypothesis-generating rather than confirmed hospital-level differences; several hospitals’ individual confidence intervals overlapped the population-level trend despite differing point estimates. This heterogeneity nonetheless indicates that a single shared temporal trend does not adequately describe hospital-level variation in ESI rates across our system, and may reflect differences in local infection prevention practices, staffing, or patient case-mix that were not captured in our analysis.

**Figure 3:**
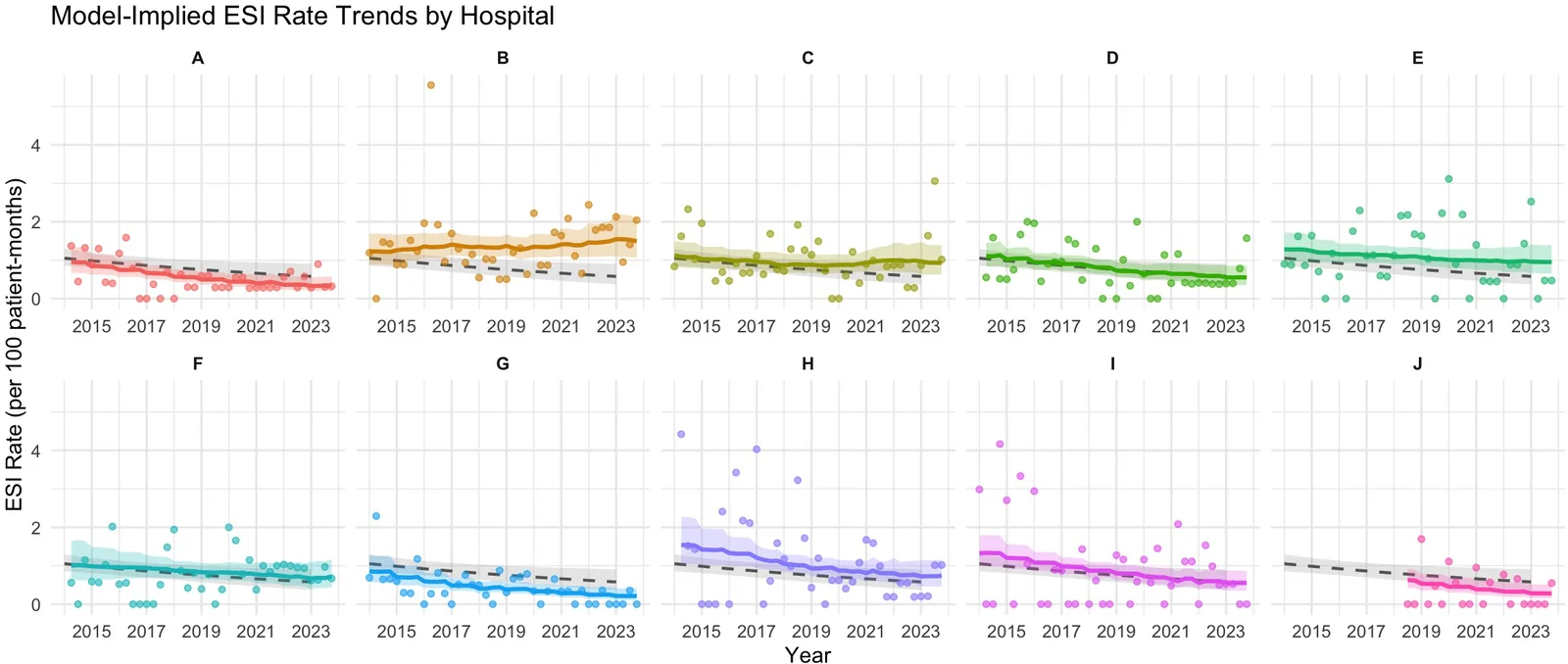
ESI rate trends by KP hospital, 2014–2023, as implied by the model. Each panel shows one KP hospital **(A–J)**. Points represent observed quarterly ESI rates (episodes per 100 patient-months). Colored lines and shaded bands show hospital-specific fitted trends with 95% confidence intervals, derived from the primary Tweedie generalized linear mixed model (random intercept and slope for calendar year by hospital), using each quarter’s observed program size. Gray dashed lines and bands show the population-level fixed-effect trend with 95% confidence interval, held constant across panels for comparison. Confidence bands for individual hospitals reflect the number of quarters observed for that hospital and should be interpreted with appropriate caution given the limited number of hospitals (*n* = 10); notably, Hospital J began reporting in 2018.

### 4.1 Limitations

As for limitations, there are considerations pertaining to the data collection process and statistical modeling. The most significant limitation of this dataset is the lack of baseline demographics of our PD cohort with ESI, which resulted in the inability to analyze differences in key characteristics such as age, sex, ethnicity, and types of PD modality over the course of the study. To address this limitation, **Table 3** demonstrates demographic data for a current cohort of PD patients from 2026, which we believe offers a representative estimation of the PD population over the course of the study. Presently, the PD population in all KPSC hospitals comprises of total of 1, 481 patients, is 61% (*n* = 903) male, and has a median of 61 years of age; racial distribution is 21.4% Asian/Pacific Islander, 13.6% Black, 46.9% Hispanic, and 15.9% White. In terms of PD modality, 32.8% and 67.2% patients were on continuous ambulatory PD (CAPD) and continuous cyclic PD (CCPD), respectively, in the 2026 KPSC PD population. Finally, while our ESI data registry collects some microorganism data, we are unable to fully characterize the ESI microorganisms due to heterogeneity of data collection methods across each KP hospital. As an approximation in 2026, of ESI cases with identified organisms, 44% and 24% were due to *Staphylococcus aureus* and *Pseudomonas aeruginosa*, respectively. ESI data are collected as aggregate rates, and our data registry does not collect microorganism data at the individual patient level. Therefore, our dataset did not allow us to correlate which ESIs were associated with peritonitis and to analyze ESI and peritonitis rates by microorganism. Future work will examine PD peritonitis rates over time and study whether this observed reduction in ESI rates may be associated with a broader trend of decreasing PD-related peritonitis rates.

**Table 3:** Demographic features of a representative PD cohort in KPSC.

Demographic	(N = 1, 481)
Age	
Range	22-94
Median	61
Sex	
Female	578 (39.0%)
Male	903 (61.0%)
Race	
American Indian/Alaska Native	2 (0.1%)
Asian/PI	317 (21.4%)
Black	201 (13.6%)
Hispanic/Latino	694 (46.9%)
White	234 (15.9%)
Unknown	33 (2.2%)
Modality	
CAPD	486 (32.8%)
CCPD	995 (67.2%)

Figures aggregated from all KPSC hospitals in 2026.

Another significant limitation surrounds methods in data collection and reporting. Program size (total patients treated), patient-months at risk, and discrete number of ESI episodes were all not available as independent quantities in our data. Consequently, we modeled ESI rate directly as a semicontinuous outcome (Tweedie distribution) rather than modeling episode counts with an offset for patient-time at risk, the latter being the more standard approach for infection rate data. Program size should therefore not be interpreted as equivalent to patient-time at risk, as duration of treatment varies by patient.

Furthermore, a small limitation of this study includes the missing quarters of data. As reported above, this missingness remained small and unclustered by hospital, but rather clustered by year; specifically, missingness concentrated to the first year, consistent with an early study ramp-up period. This explains the effects visible in the missing data matrix (**Supplementary Figure 1A**). A sensitivity analysis excluding the center with delayed reporting produced materially consistent estimates (**Supplementary Table 2**), supporting the robustness of our findings to this source of incompleteness.

Our model’s random-effects structure is also constrained by the number of centers available (*n* = 10). While our fixed-effect estimates for calendar year and program size are well-supported by the full longitudinal panel, changes in ESI rate for specific KP hospitals carry greater uncertainty and should be interpreted cautiously, as discussed above. Finally, as an observational analysis without patient-level or demographic data, our findings establish a temporal association rather than a causal mechanism, and generalizability beyond this single integrated health care system should be hypothesized cautiously.

## 5 Conclusion

PD is a promising treatment modality for patients with ESKD. Evidence from KPSC’s ten hospitals demonstrates that ESI rates from PD have been decreasing in a statistically and clinically significant manner over a ten-year period. This decline was not explained by growth in program size, suggesting that other factors—such as evolving infection prevention practices, staff training, or care coordination—may have contributed, though our data do not permit us to test these mechanisms directly. Future work will include mechanism-specific studies to explore which factors are driving these improvements as well as more streamlined data collection to associate changes in ESI rates with peritonitis rates and microorganism data. The results of this study encourage the continued implementation and refinement of PD at-scale and raise the possibility that greater cumulative experience with this modality may contribute to improved outcomes for ESKD patients on PD.

## Data Availability

The raw deidentified data supporting the conclusions of this article will be made available by the authors, without undue reservation.
