# Supplementary material for: Improvement in peritoneal dialysis exit-site infection rates in an integrated health care system in the United States
Source: Front Nephrol. 2026 Sep 17;6:1825353. doi: 10.3389/fneph.2026.1825353 (PMC13626983; doi:10.3389/fneph.2026.1825353)
Supplement: Supplementary file 1 [file SupplementaryFile1.docx]

Supplementary Material

# 1 This supplement provides additional detail on mathematical reasoning, data coverage, missing data patterns, model selection, and sensitivity analyses supporting the primary results presented in the main text. Supplemental Tables S1–S2 and Supplemental Figures A–B are referenced at the relevant points in the Methods, Results, and Discussion sections throughout.

# 2 Supplemental Results

# 3 *Model selection and diagnostics.* A model with a random intercept and slope for calendar year by center fit significantly better than a random-intercept-only model (likelihood ratio test, *χ*²=11.3, df=2, *p*=0.004) and resolved residual non-uniformity present in the simpler model (Kolmogorov-Smirnov test on scaled residuals: *p*=0.020 for the random-intercept-only model versus *p*=0.25 for the final model). The final model showed no evidence of overdispersion (*p*=0.19) or inadequate zero-mass capture (*p*=0.47) on DHARMa diagnostic testing (Supplemental Figure B). Variance components indicated meaningful between-center heterogeneity in both baseline ESI rate (intercept SD=0.19) and rate of decline over time (slope SD=0.06; intercept-slope correlation=0.36). Center-specific model-implied trends are shown in Figure 3; most centers showed declining trends broadly consistent with the population-level estimate, though at least one center showed a trend in the opposite direction. Given the limited number of centers (*n*=10), center-specific estimates should be interpreted with appropriate caution.

# 4 *Model results.* See Table 2 of main text for calendar year and program size increases. The intercept (baseline rate, 2014) had an IRR of 0.014 (0.010–0.019 95% CI). The intercept represents the model-implied ESI rate in 2014 at a program size of zero patients, a mathematical reference point required for model parameterization rather than a directly interpretable clinical quantity; it should not be read as the typical 2014 rate at observed program sizes (see Table 1 for descriptive 2014 rates). Random-effects variance components indicated meaningful between-center variability in both baseline ESI rate (intercept SD = 0.191, on the log scale) and rate of decline over time (Year slope SD = 0.063, on the log scale), with a moderate positive correlation between the two (r = 0.36), suggesting that centers with higher baseline ESI rates tended to show somewhat steeper declines over the study period.

# 5 *Sensitivity analyses.* A model incorporating a nonlinear (quadratic) term for program size did not improve fit over the linear specification (ΔAIC<1; likelihood ratio test, *p*=0.084) and was not retained (Supplemental Table S1). Excluding the center with delayed reporting did not materially change primary conclusions: the temporal decline remained significant (IRR 0.95, 95% CI 0.90–1.00, *p*=0.036) and program size remained non-significant (IRR 0.71, 95% CI 0.49–1.04, *p*=0.076) (Supplemental Table S2).

# 6 *Internal validity.* The magnitude of decline estimated by our longitudinal model is consistent with the pattern observed in unadjusted descriptive statistics: mean ESI rates fell from 1.26 to 0.72 episodes per 100 patient-months between 2014 and 2023, a 43% relative decrease, closely matching the 45.1% cumulative decline (95% CI: 12.8–65.4%) estimated by our primary model over the same period. This concordance between a purely descriptive comparison and a fully adjusted longitudinal model provides reassurance that our model-based estimate reflects a genuine feature of the data rather than an artifact of model specification.

# 7 *Future modeling directions.* Our model captures temporal trends through a random slope for calendar year rather than an explicit time-series structure; future work could explore formal autocorrelation modeling, such as autoregressive integrated moving average (ARIMA) approaches or Bayesian hierarchical time-series frameworks, to further characterize temporal dynamics in ESI rates.

# 8 Supplementary Figures and Tables

## 8.1 Supplementary Figures

**Supplementary Figure A.** Data availability matrix depicting the presence or absence of data at each KP hospital for every quarter in the period of study. Green indicates data present; orange indicates location-quarters with recorded rows but missing program size and denominator data ("dropped data," *n*=7); gray indicates location-quarters prior to a center's reporting start or the 2013 Q3–Q4 registry-wide administrative gap ("predates tracking"). The blue underline denotes the period included in all models (2014 Q1–2023 Q4).

**Supplemental Figure B.** Residual diagnostics for the random-intercept-only model (A, top) versus the final random-slope model (B, bottom), from simulation-based residual checks using DHARMa, a package for residual diagnostics in hierarchical (multi-level) mixed regression models. Each panel set shows a QQ plot of scaled residuals (left) and residuals versus model predictions (right). The random-intercept-only model showed evidence of residual non-uniformity (Kolmogorov-Smirnov test, *p*=0.020) and a significant deviation in the residuals-versus-predictions test, localized to the program-size predictor; both were resolved in the final model (KS test, *p*=0.25). Dispersion and outlier tests were non-significant in both models.

## 8.2 Supplementary Tables

**Supplemental Table S1.** Model comparison: random-intercept-only, random-slope, and quadratic program-size specifications. Akaike Information Criterion (AIC), Bayesian Information Criterion (BIC), and likelihood ratio test results are shown relative to the random-intercept-only baseline. None of the three models showed evidence of a singular fit, indicating that the added complexity of the random-slope specification was well-supported by the available data rather than reflecting overparameterization

| Model | AIC | BIC | Singular |
| --- | --- | --- | --- |
| Random-intercept-only (baseline) | -2004.37 | -1980.81 | FALSE |
| Quadratic program size (sensitivity check) | -2005.35 | -1977.86 | FALSE |
| Random slope for Year (final model) | -2011.64 | -1980.23 | FALSE |

**Supplemental Table S2. Sensitivity analysis excluding the hospital with delayed reporting (J).** Fixed-effect estimates for calendar year and program size are shown for the full panel (n=10 hospitals) and after excluding J (n=9 hospitals). Estimates were similar in direction and magnitude with and without J, indicating that the primary findings were not driven by this hospital’s delayed entry into the panel.

| Model | Term | Log-Scale Estimate | IRR (95% CI) | *p*-value |
| --- | --- | --- | --- | --- |
| Full panel (n=10 hospitals) | Calendar Year | -0.067 | 0.936 (0.889–0.985) | 0.011 |
|  | Program Size (per 100 patients) | -0.281 | 0.755 (0.517–1.105) | 0.148 |
| Excluding J (n=9 hospitals) | Calendar Year | -0.055 | 0.947 (0.899–0.996) | 0.036 |
|  | Program Size (per 100 patients) | -0.339 | 0.712 (0.490–1.040) | 0.076 |
